# Aducanumab: una mirada dos años después de su aprobación

**DOI:** 10.7705/biomedica.6967

**Published:** 2024-05-31

**Authors:** Astrid Torres, Loida Camargo, Norman López

**Affiliations:** 1 Programa Neurología Clínica, Universidad del Sinú, Cartagena, Colombia Universidad del Sinú Universidad del Sinú Cartagena Colombia; 2 Grupo de Investigación Neurociencia y Salud Global, Facultad de Medicina, Universidad de Cartagena, Cartagena, Colombia Universidad de Cartagena Universidad de Cartagena Cartagena Colombia; 3 Escuela de Kinesiología, Facultad de Salud, Universidad Santo Tomás, Santiago, Chile Universidad Santo Tomás Universidad Santo Tomás Santiago Chile; 4 Centro de Investigación en Gerontología Aplicada (CIGAP), Facultad de Salud, Universidad Santo Tomás, Santiago, Chile Universidad Santo Tomás Universidad Santo Tomás Santiago Chile

**Keywords:** Alzheimer’s disease, β-amyloid, disease-modifying therapies, aducanumab, enfermedad de Alzheimer, β amiloide, terapias modificadoras de la enfermedad, aducanumab

## Abstract

Alzheimer’s disease is the leading cause of dementia worldwide and a critical public health problem. While deaths from cardiovascular diseases have decreased, those attributed to Alzheimer’s disease have increased in recent years with no curative treatment to date. In this context, effective treatment development has become a global priority. Aducanumab is a human anti-amyloid β monoclonal antibody approved by the FDA in June 2021 for the treatment of Alzheimer’s disease but failed to show the expected clinical efficacy in phase III trials.

This review analyzes the history of its controversial acceptance, implications, and prospects for future treatment.

## Resumen

La enfermedad de Alzheimer es la principal causa de demencia en todo el mundo y representa un importante problema de salud pública. Si bien las muertes por enfermedades cardiovasculares han disminuido, las atribuidas a la enfermedad de Alzheimer han aumentado en los últimos años y hasta la fecha no existe tratamiento curativo. Por este motivo, el desarrollo de un tratamiento eficaz se ha convertido en una prioridad mundial. Aducanumab es un anticuerpo monoclonal anti-amiloide β humano aprobado para el tratamiento de la enfermedad de Alzheimer en junio de 2021 por la FDA, sin la eficacia clínica esperada en los ensayos de fase III. Esta revisión analiza la historia de su controvertida aceptación, implicaciones y perspectivas para el tratamiento futuro.

Alzheimer’s disease is a neurodegenerative disorder characterized by predominantly amnesic progressive cognitive impairment, behavioral changes, and loss of functional abilities ^1^. It is the leading cause of dementia worldwide and a critical public health problem ^2^.

According to the Alzheimer’s Association, 6.5 million Americans had the disease in 2022, and the number of affected people aged 65 and older is expected to reach 13.8 million by 2060. Between 2000 and 2019, deaths from cardiovascular diseases decreased by 7.3%, while those attributed to Alzheimer’s increased by 145% ^3^. Various therapeutic strategies have been explored, but to date, there is no curative treatment.

By 2003, only four drugs had been approved by the Food and Drug Administration (FDA). Three of them are inhibitors of the acetylcholinesterase enzyme (donepezil, galantamine, and rivastigmine), and one is a noncompetitive antagonist of the NMDA glutamate receptor (memantine). They all show a discrete symptomatic improvement but none in cognition ^4^.

In this context, disease-modifying therapies have become a global priority. Considering that the accumulation and aggregation of β-amyloid is the main cause of the neurodegenerative process, promoting its elimination through specific anti-e-amyloid antibodies is a rational intervention. Immunotherapy targeting β-amyloid has so far been the most promising strategy to delay Alzheimer’s disease progression ^1^.

## Historical count

Aducanumab -a fully human monoclonal antibody- has generated the highest expectations in recent years. Neuroimmune^TM^ was the first to manufacture it, and later, in 2007, Biogen^TM^ bought the license ^5^. After a series of discussions, on June 7, 2021, the FDA granted it conditional approval as the first disease-modifying agent ^6^ but recommended further studies. However, most of the FDA advisory board members voted against it. The European Medicines Agency recently disapproved of its use in European countries ^7^.

This review analyzes the background of its controversial acceptance, implications, and perspectives for future treatment.

Initially, aducanumab was indicated for patients at any stage of Alzheimer’s disease, although only patients with mild disease were included in the clinical trials. In an updated indication, the FDA limited its use to the type of population included in the trials.

After an initial titration period, a 10 mg/kg dose of aducanumab should be administered as a monthly intravenous infusion. Once absorbed, it reached a maximum concentration of 182.7 μg/ml with a maximum time of three hours and a lower area under the curve of 31,400 h x μg/ml. After 16 weeks, researchers saw stable concentrations and a mean distribution volume of 9.63 L. The clearance was 0.0159 L/h, and the terminal half-life was 24.8 days ^8^.

PRIME was a small phase 1b clinical trial with 165 patients, designed by Biogen, to evaluate the effect of different aducanumab doses (1, 3.6, and 10 mg/kg) on β-amyloid elimination ^6^. The first results, published in 2016, showed a dose- and duration-dependent reduction in amyloid plaque and a possible slowing of cognitive deterioration ^9^.

Patients receiving the highest dose had a higher β-amyloid elimination rate. However, structural imaging amyloid-related alterations such as edema and hemorrhage also occurred more frequently in this group, especially among patients carrying the gene *APOE4*^6^.

After these promising results, in 2015, the pharmaceutical company Biogen conducted ENGAGE and EMERGE, two phase 3 clinical trials, including 3,285 patients with mild cognitive impairment or early dementia from different centers and countries ^10^.

These methodologically identical trials compared low and high drug doses versus placebo in patients with an average age of 70 years, of whom two- thirds were carriers of the *APOE4* allele. In March 2019, both studies were stopped upon meeting pre-established futility criteria for interim analyses based on data from the first 1,748 patients ^11^.

In October 2019, Biogen announced that it would ask the FDA for approval based on the analysis of additional data from EMERGE demonstrating a significant effect on cognitive and functional impairment in patients treated with the highest dose ^12^. Patients receiving higher doses had a 22% decrease in adjusted mean clinical dementia rating scores on the Clinical Dementia Rating-Sum of Boxes questionnaire ^13^. However, the difference in absolute terms was 0.39 points, far from what it considered clinically relevant in 1-2 points trials ^14^.

The most common adverse events reported in these two studies were amyloid-related radiological neuroimaging abnormalities, headache, diarrhea, and falls. Cerebral edema was reported in 34 and 35.5% of patients receiving high doses of aducanumab in EMERGE and ENGAGE, respectively ^6^.

In November 2020, an FDA advisory committee analyzed the data presented by Biogen, issuing a negative report with ten votes against and one abstention due to no compelling evidence of cognitive improvement in patients treated with aducanumab. They also considered high cost of the treatment and the previously documented side effects ^7^.

Despite conflicting results from these two identically designed studies and the lack of evidence of β-amyloid reduction correlated with clinical improvement, in April 2021, an internal FDA board determined that aducanumab would meet the bar for accelerated approval since its effect on β-amyloid plaques could slow Alzheimer’s disease progression ^15^.

It is worth noting that this approval route has previously been applied to other drugs for diseases such as cancer, AIDS, or, more recently, COVID-19, but always when confirmatory efficacy trials are underway. In the case of aducanumab, both trials had been suspended due to futility. Not even in EMERGE Biogen could show the association between amyloid load elimination and clinical efficacy ^7^.

## Aducanumab use in real life

In *post hoc* analysis of trial data, aducanumab did not reverse cognitive dysfunction. Patients only showed a modest degree of protection against the progression of cognitive decline in a subset of subjects who received the highest dose of medication ^6^.

Some experts opposing aducanumab approval argued higher associated risks than benefits attributed to the drug. For example, dose-dependent transient amyloid-related structural alterations may outweigh slight improvement in cognitive impairment ^13^.

Salloway *et al*., in their study on the adverse effects of different treatment schemes, demonstrated that after completing 20 doses of aducanumab, 41% of the patients presented amyloid dose-dependent radiological alterations such as edema, superficial siderosis, and microbleeds, the latter especially frequent in elderly patients ^16^.

Moreover, the drug is considered unprofitable, with an annual acquisition cost of USD $56,000 that further increases due to screening and follow-up testing (PET imaging and periodic magnetic resonance imaging), APOE4 genotype assays, and the cost related to drug administration every four weeks ^17^.

On the other hand, the populations included in these trials were not ethnically diverse, without adequate representation of many groups, including blacks, Hispanics, and indigenous people. In this sense, drug safety and effectiveness in these populations is unknown ^10^.

The Alzheimer’s Association accepted aducanumab approval with the initial indication, while the consumer association Public Citizens rejected it after requesting the abandonment of three FDA positions involved in the accelerated approval ^7^.

The controversy reached such magnitude in the United States that two House of Representatives committees in Washington are carrying out an investigation into the approval process and the drug price. The head of the FDA has asked the Department of Health and Human Affairs for an independent investigation into the approval of aducanumab ^18^.

Contrary to expectations, most drugs targeting β-amyloid have not demonstrated efficacy in late-stage clinical trials. It raises concerns about patients dropping other drug trials just to be treated with aducanumab, which diverts attention from molecule development targeting other therapeutic alternatives ^7^.

In recent years, interest in tauopathies has increased, with favorable preclinical results in the first stages of underway clinical trials ^1^. Some researchers suggest these diseases could play a primary role in Alzheimer’s pathogenesis, as the severity of cognitive impairment correlates better with the accumulation of tau than β-amyloid ^19^.

Finally, based on the precedent of aducanumab, the accelerated approval route may be justified for drugs with similar action mechanisms now being analyzed. It calls for weighing the biological plausibility criterion over the expectations of the scientific community, patients, and society in an era where trust is placed in the autonomy of regulatory agencies.
